# Virtual Reality Gaming and Its Impact and Effectiveness in Improving Eye–Hand Coordination and Attention Concentration in the Oldest-Old Population

**DOI:** 10.3390/jcm14134651

**Published:** 2025-07-01

**Authors:** Żaneta Grzywacz, Justyna Jaśniewicz, Anna Koziarska, Dorota Borzucka, Edyta Majorczyk

**Affiliations:** 1 Faculty of Production Engineering and Logistics, Opole University of Technology, 76 Prószkowska St., 45-758 Opole, Poland; j.jasniewicz@po.edu.pl; 2 Center of Education Applications of Mathematic, Opole University of Technology, 76 Prószkowska St., 45-758 Opole, Poland; a.koziarska@po.edu.pl; 3 Faculty of Physical Education and Physiotherapy, Opole University of Technology, 76 Prószkowska St., 45-758 Opole, Poland; d.borzucka@po.edu.pl

**Keywords:** virtual reality (VR), eye–hand coordination (EHC), attention concentration (AC), the oldest-old population, rehabilitation

## Abstract

**Background**: The ageing process is associated with a decline in cognitive functions, including eye–hand coordination, attention concentration, and psychomotor reaction time. This study aimed to assess the effectiveness of virtual reality–based therapy in enhancing cognitive functions in seniors. **Methods**: This study was conducted on 38 cases (29 women and 9 men) with a mean age of 87.2 years, who were divided into two groups: a VR group (with a 4-week, three-time-week training program using the game “*Beat Saber*”) and a control group (with a standard 4-week exercise program). Assessments of eye–hand coordination and attentional concentration were conducted at the beginning (T_0_) and the end (T_1_) of the training. **Results**: Analysis of eye–hand coordination and attentional concentration showed significant improvement in both groups (T_0_ vs. T_1_: *p* = 0.0002 for the intervention group and *p* = 0.007 for the control group). However, the effect in the VR group was almost three times greater than in the control group (1.689 vs. 0.615 in D effect). Moreover, in the VR group, an analysis of “good cuts” indicated improvements in both parameters after 4 weeks of VR training. The percentage of correctly received stimuli increased significantly across sessions (*p* < 0.00001). Furthermore, 84.3% of participants experienced a twofold improvement in performance over the 12 VR sessions (42% vs. 80% accuracy in successful hits). The distribution of results also suggests a positive subjective impact of VR therapy in maintaining mental activity. **Conclusions**: The findings indicate that VR-related training can support elderly individuals in recovering cognitive function, potentially enhancing their independence and life quality.

## 1. Introduction

Technological progress plays a significant role in the advancement of medicine and healthcare. Meanwhile, in public health, knowledge about a healthy lifestyle (physical activity and nutrition) continues to expand. As a result, life expectancy is rising worldwide, leading to a growing ageing population. In 2020, the number of people aged 60 years and older exceeded the number of children younger than 5, and the global population over 60 years old is expected to nearly double, rising from 12 to 22%. It is predicted that by 2030, one in six people in the world will be 60 years old or older, and by 2050, the global population of people aged 60 and over will double, reaching 2.1 billion. Moreover, the number of people aged over 80 is expected to triple between 2020 and 2050 [1]. A similar trend is forecast for Polish society, indicating that by 2060, the number of people aged 65 and over will increase by 34%, and the population aged over 80 will more than double compared to 2022 [2].

Older people often experience a decline in cognitive function, which encompasses various dimensions, including attention, orientation, working memory, visuospatial ability, executive function, and processing speed [3]. Consequently, a deterioration in quality of life (QoL) often occurs, making it essential to find a way to improve seniors’ QoL. In this context, it is particularly important to meet the basic needs, such as health, safety, independence, and social participation, as they are crucial determinants of QoL in older adults. Conversely, unmet needs in areas such as access to healthcare, social support, and opportunities for active leisure can significantly diminish seniors’ QoL [4,5]. Moreover, from a functional perspective, one of the key factors influencing QoL is eye–hand coordination (EHC), which impacts the ability to perform activities of daily living. Cognitive decline negatively affects EHC, leading to issues such as reduced muscular endurance and poor coordination [6,7,8]. Proper EHC is crucial for maintaining seniors’ independence, preventing falls, supporting cognitive function, as well as promoting greater social engagement. Impairments in EHC can lead to a rapid loss of independence and, consequently, contribute to a further decline in QoL [9,10].

EHC refers to the control of eye and hand movement and the process through which they work together. EHC is regulated by the cerebral cortex, which triggers the hand muscles to perform movement at the most appropriate time [11,12]. EHC consists of three key sub-abilities: visual–motor integration, visual perception, and motor coordination [13], which contribute to proper EHC, which in turn can help maintain greater independence in older adults [14].

Due to these factors, effective interventions that can slow or prevent cognitive function deterioration, including strategies to mitigate the deterioration of EHC, are crucial for older adults [12,15,16,17,18,19]. Therefore, various therapies have been proposed to enhance EHC in seniors. For instance, promising results were obtained with brain gym exercises [20], interactive cognitive-motor training [18], and horticultural activities [19]. Additionally, studies confirm that video games have a positive impact on cognitive and emotional functions in early adulthood [21], but for older individuals, this type of solution may be extensively complex.

In this context, emerging technologies offer new possibilities, providing simpler and more engaging therapeutic solutions for seniors. High-quality head-mounted display–based virtual reality (HMD-VR) has generated significant interest, as it is becoming more widely available and can serve as an innovative supportive intervention. HMD-VR health games are tools for engaging users in highly immersive experiences to promote health and well-being [22]. For example, the game “*Beat Saber*” is a unique VR rhythm game that encourages physical activity by requiring players to match their movements with a musical beat. Achieving high scores and effectiveness in the “*Beat Saber*” relies on appropriate psychomotor skills, including visual–motor coordination and cognitive functions. This suggests that such games could serve as accessible and effective therapeutic tool for seniors. Therefore, the aim of this study was to assess the impact of VR-based training on eye–hand coordination and attention concentration in seniors. We investigated whether the utilization of the commercial immersive game “*Beat Saber*”, which provides light stimuli, may improve EHC and AC in the study group.

## 2. Materials and Methods

### 2.1. Participants

Thirty-eight elderly people (29 females and 9 males; aged 65 and older) were enrolled in this study (the recruitment period from 17 July 2024 to 19 July 2024). Participants were randomly assigned to one of two groups: the VR (intervention) group or the control group, using Research Randomizer (https://www.randomizer.org/, accessed on 19 July 2024). Randomization was performed in a 1:1 ratio and stratified by sex (male and female).

All participants lived in a Nursing Home in Opole for an average of 2 years and had similar nutritional habits and daily activity levels. Therefore, participants from both the VR and control groups followed a physical activity program offered by the institution. The program was designed to improve overall physical fitness and coordination. Each exercise session included a warm-up, general fitness exercises using simple equipment (e.g., gymnastic bands, sticks, and dumbbells), coordination exercises (e.g., exercises with music and rhythm and catching and throwing the ball), and finally stretching and relaxation exercises. The program condition was adapted to the abilities of seniors, supervised by a physiotherapist, and additionally fostered social participation.

Eligibility was determined by a physician who assessed each participant’s health condition and screened for contraindications to HMD-VR use (in the VR group). Inclusion criteria included being aged 60 years or older, having good overall fitness, and signing an informed consent to participate in this study. Exclusion criteria included lack of consent, contraindications to VR headset using, such as current temporary conditions (e.g., fatigue or exhaustion, drowsiness, nausea, anxiety or stress, cold, and flu) and chronic conditions (e.g., cancer in an intensive treatment phase, inability to exercise, visual or auditory disorders preventing VR goggles use, and cognitive disorders that hindered understanding exercise instructions or cooperating with the researcher). For characteristics, see Table 1.

**Figure 1:**
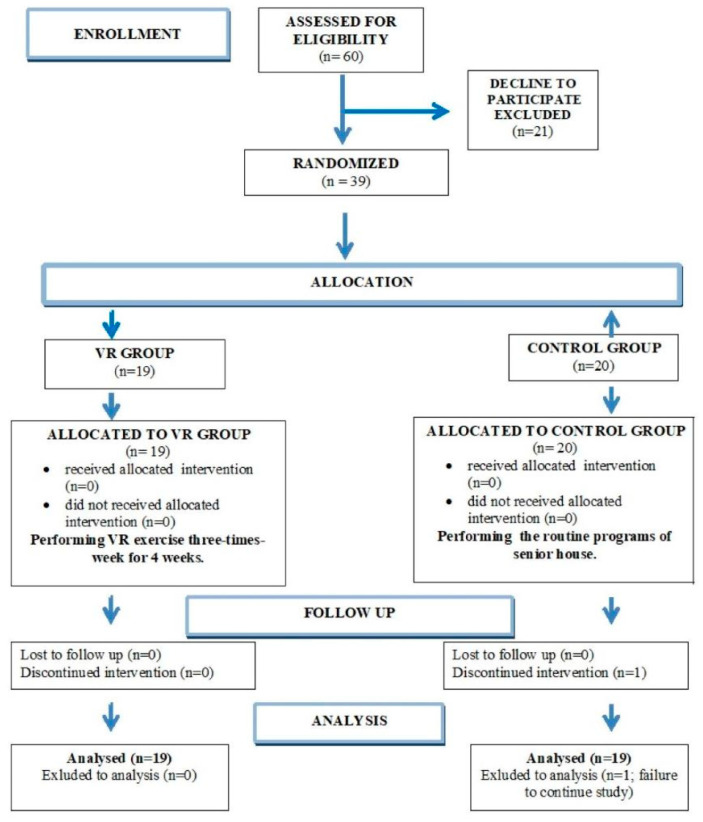
CONSORT 2010 flow diagram. The diagram presents the flow of participants through each stage, including enrollment, allocation, follow-up, and analysis. A total of 60 individuals were assessed for eligibility, of whom 39 met the inclusion criteria and were randomized. The main reasons for exclusion was a decline in participation (*n* = 21). During the follow-up stage, 1 participant was excluded, and an analysis was performed on 38 cases (19 in the VR group and 19 in the control group).

This study was conducted in accordance with the Declaration of Helsinki and was approved by the Bioethics Committee of the Hirszfeld Institute of Immunology and Experimental Therapy (approval number: KB-10/2024). Written informed consent was obtained from all participants. This study was designed and reported following the CONSORT 2010 guidelines (Figure 1).

### 2.2. Study Design Intervention

**Figure 2:**
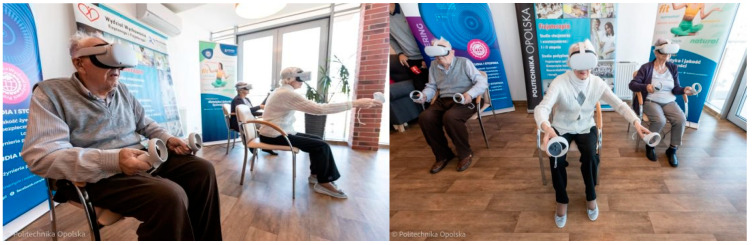
Seniors during VR-based intervention (source: Opole University of Technology gallery).

An introductory session was organized to familiarize seniors with the VR. Then, a 4-week training program was conducted for participants in the VR group, consisting of 12 sessions (three times per week), each lasting 20 min. All sessions were performed in a seated position. The training program was based on the popular rhythm game “*Beat Saber*”, featuring songs by Queen (e.g., “We will rock you”, “We are the champions”, and “Another one bites the dust”), each in varying rhythms and tempos. During gameplay, participants received visual stimuli (lights) and were required to cut blocks in sync with the music using either their right or left hand as indicated. Each block had to be sliced with a lightsaber of the corresponding color. For user comfort, the game was preloaded onto the VR headset. The VR source consisted of a Meta Quest 2 headset (Meta Platforms, Menlo Park, CA, USA), with goggles and intuitive controllers equipped with sensors. Moreover, the VR glasses were synchronized with portable screens, allowing researchers to monitor gameplay and assist participants. Before starting each session, the participant’s headset was adjusted to the anthropometric dimensions of their head, and corrective glasses were provided if needed. The VR-related exercises were conducted across 3 game areas; each game area had no walls or arrows and was individually marked using motion sensors, covering approximately 4 m^2^. For the safety of elderly participants, heart rate (HR) and energy expenditure (EE) were monitored during the sessions (Fitbit Charge 6 by Google). Oxygen saturation was also measured before and after exercises (Figure 2). Additionally, after the VR-based intervention, participants were asked to provide subjective feedback regarding the helpfulness of the therapy in mental activity. They could respond to a closed-ended question using one of the following options: “definitely yes”, “probably yes”, “hard to say”, “probably no”, or “definitely no”. The control group was a passive group that did not receive any specific interventions. This group only participated in standard exercises conducted daily at the senior home.

### 2.3. Outcome Measures

Assessments were performed at the beginning (T_0_) and at the end of the corresponding training, i.e., after 4 weeks (T_1_; 20 min after the last session). In the VR group, all sessions were monitored for HR, EE, and the game effectiveness.

#### 2.3.1. Eye–Hand Coordination and Attention Concentration Measurements (T_0_/T_1_)

The cross visual–motor test using the Piórkowski apparatus (PIOR/ATB 2.0) was used to assess EHC and AC. The reaction times and movement accuracy errors with millisecond precision were automatically recorded by the device. During the test, participant was seated comfortably in front of the apparatus with their hands positioned over the buttons. The following test pattern was used: a duration of 90 s, with 1-second light intervals, and stimulus frequency of 90 stimuli per 90 s. On the device’s panel, lights were illuminated in random sequences, and the participant had to respond immediately by pressing the corresponding button as quickly and accurately as possible. The device recorded the subject’s movements, reaction times, and errors in each light stimulus. The test was conducted over three consecutive trials, and then average score was calculated for each metric.

#### 2.3.2. Control of the Effectiveness of “Good Cuts” During a Gaming in the Intervention Group

During all gaming sessions, the number of “good cuts”—correct reactions to lights (cutting the square blocks using color-matched lightsabers using right or left hand in sync with the music) was monitored. Points obtained in each round of the VR physical activity session were recorded, and the results from individual rounds were summed and compared with the maximum possible score.

### 2.4. Statistical Analysis

Demographic characteristics of the analyzed groups were presented using descriptive statistics (mean and standard deviation), and the Mann–Whitney U test was used to evaluate differences between them. Additionally, descriptive statistics were used for the game results (“good cuts”), and the Friedman ANOVA test was used to analyze changes in game performance across subsequent sessions. For EHC and AC, normality test was performed using the method of Shapiro–Wilk, and according to the data, non-normal distribution, Mann–Whithey U (for intergroup analysis), and Wilcoxon (for intragroup and intratrial analyses) tests were used for statistical analysis. Herein, *p*-values ≤ 0.05 were considered significant. Statistical analysis was performed using Statistica v.13 (TIBCO Software Inc., Palo Alto, CA, USA).

## 3. Results

**Figure 3:**
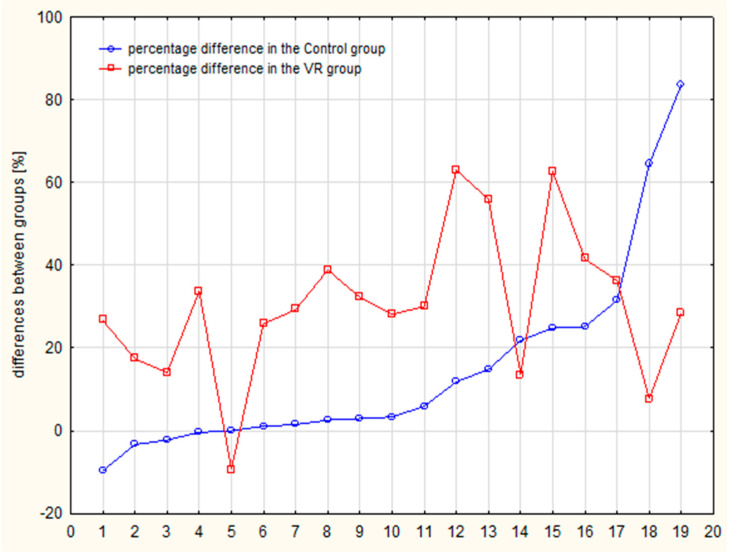
Individual differences in the cross visual–motor test for EHC and AC analysis. Individual differences for each of 38 participant: 19 in the VR group (red; 15 participants with meaningful improvement and 1 person #5 with significant deterioration of EHC and AC) and 19 in the control group (blue; 1 person with improvement and 4 with deterioration in EHC and AC).

The results were obtained from a group of 38 elderly participants divided into two groups: the VR group (N = 19) and the control group (N = 19). The presented results were collected from participants in accordance with the CONSORT flow (Figure 1). The VR-based intervention was carried out without any reported adverse effects or interruptions, and in adherence to medical recommendations—all cases fulfilled the intervention. Only two participants reported discomfort (visual disturbances and dizziness); however, these did not affect the continuation in the intervention. For the analysis of EHC and AC in the context of the VR physical activity, all participants completed three trials of the cross visual motor test at T_0_ (before intervention) and at T_1_ (after intervention). As shown in Table 2, statistically significant results were obtained in both intergroup and intragroup analyses. In the intragroup analysis of the VR group, when results at T_0_ and T_1_ were compared, a 30.4% increase was observed (45.1% vs. 75.5% at T_0_ and T_1_, respectively). The mean difference between time points was large (d = 1.689; *p* < 0.0002, paired Wilcoxon test), indicating a substantial effect. In the intragroup analysis of the control group, a significant increase in obtained means was also noted (17.3% at T_0_ and 32.0% at T_1_, difference of 14.7%). In this analysis, the paired Wilcoxon test showed *p* = 0.0065 and d = 0.615. These results indicated that EHC and AC were improved in the VR group nearly three times more strongly than in the control group. In turn, intergroup comparisons between the VR group and the control group showed significant differences at both time points (45.1% vs. 17.1% and *p* = 0.0003 at T_0_ and 75.5% vs. 32.0% and *p* = 0.0003 at T_1_). In these U-Mann–Whitney tests, the effect size was also higher after the 4-week training period (1.692 vs. 1.281 at T_0_), suggesting that the VR-related intervention significantly improved EHC and AC compared to the standard training program in the control group. Moreover, the individual results of seniors were analyzed, showing that 15 of 19 participants in the VR group and only 1 case in the control group achieved meaningful improvement. On the other hand, four participants in the control group and one person in the VR group showed deterioration of EHC and AC results (Figure 3).

**Table 2:** Intergroup and intragroup differences in eye–hand coordination and attention concentration.

Cross Visual Motor Test (% of 90 Light Stimulus)	Groups			
	VR Group T_0_ (Before)	VR Group T_1_ (After)	Control Group T_0_ (Before)	Control Group T_1_ (After)
Mean (SD)	45.1 (21.6)	75.5 (15.4)	17.3 (21.8)	32.0 (32.9)
Median (Q_1_–Q_3_)	42.2 (28.9–64.8)	77.4 (66.3–88.5)	8.5 (3.3–19.3)	9.6 (4.8–69.3)
**Statistical analyses**				
**Test**	**Comparison**		***p* value**	**D effect**
U Mann Whitney	VR group in T_0_ vs. control group in T_0_		0.0003	1.281 *
	VR group in T_1_ vs. control group in T_1_		0.0003	1.692 *
Paired Wilcoxon	VR group in T_0_ vs. VR group in T_1_		0.0002	1.689 **
	control group in T_0_ vs. control group in T_1_		0.0065	0.615 **

$$(*)d=\frac{\bar{X_{1}}-\bar{X_{2}}}{s}\;\;(**)\;d=\frac{\bar{D}}{s_{D}},\;\mathrm{where}\;D=\bar{X}\mathrm{after}\;-\;\bar{X}\mathrm{before}$$

**Figure 4:**
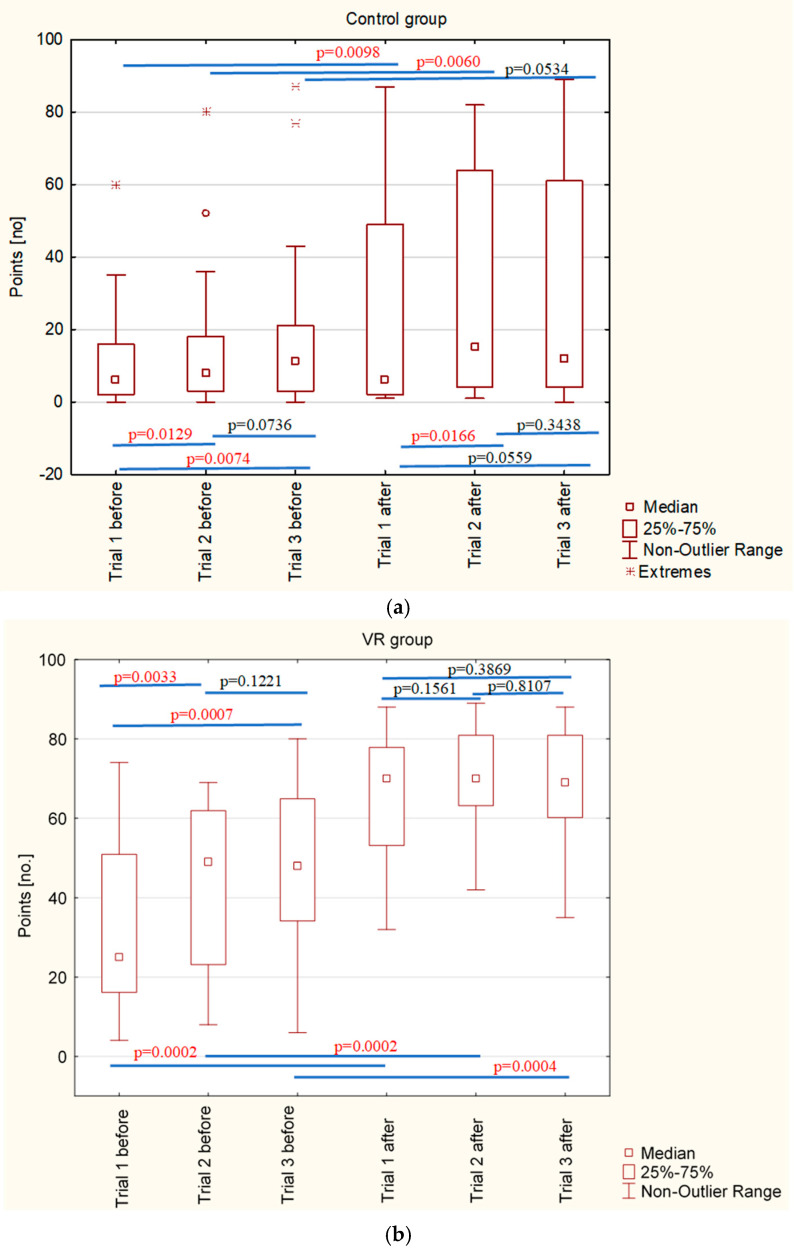
Learning effect across consecutive trials of the cross visual–motor test for ACH and AC; (**a**) control group before and after the 4-week standard program at the senior house, and (**b**) VR intervention group before and after the 4-week VR-related training. Box-and-whisker plots showing performance across three trials, before and after 4-week training. A noticeable improvement was observed between trial 1 and trial 2, with further improvement or stabilization in trial 3. Differences between trials were more pronounced before the 4-week training than after, suggesting a learning effect and increased consistency after intervention.

As mentioned above, all cases completed three trials of the ACH and AC test at both time points. It was observed that participants showed significant improvement across the three trials, indicating learning effects over time. There was a noticeable improvement in performance from trial 1 to trial 2, and additional improvement or maintenance was observed in trial 3. The differences between individual trials were greater before 4-week training than after (Figure 4a,b).

**Figure 5:**
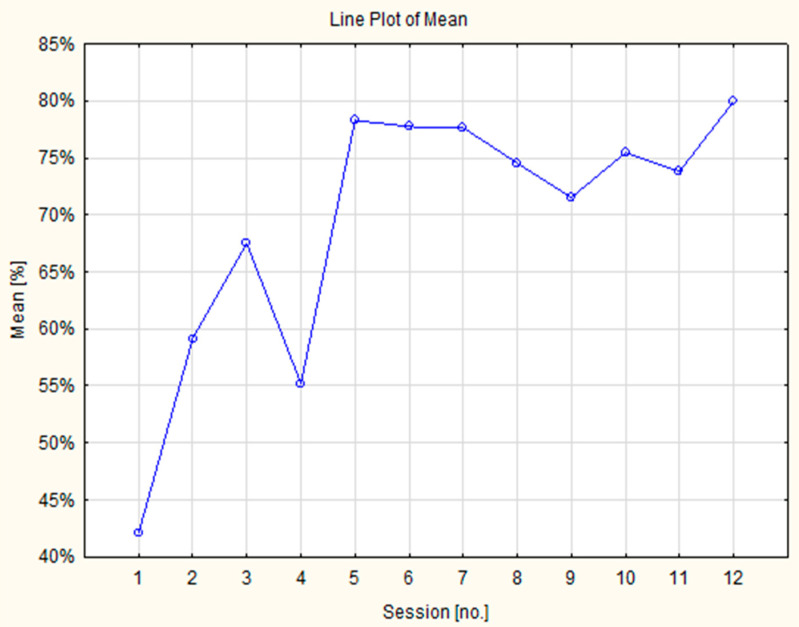
Results of the effectiveness of the game “*Beat Saber*” (“good cuts”) in consecutive sessions of VR-related training. Line graph showing participants’ performance across 12 training sessions. A general upward trend (the average of “good cuts” increased across 12 sessions from 42% to 80%) indicates progressive improvement over time, suggesting the effectiveness of the training program.

Additionally, the gaming performance of the VR group was analyzed. Using the music immersive game “*Beat Saber*”, the number of “good cuts” (correctly cutting square blocks, i.e., with color-matched lightsabers using the right or left hand in sync with the music) was recorded across 12 training sessions (Figure 5). The mean number of “good cuts” doubled from 42% in session 1 to 80% in session 12, with a significant difference in performance across sessions (*p* < 0.00001). Interestingly, in session 12, the lowest individual result was 50% effectiveness, achieved by a senior with mild paresis of the left hand. Moreover, VR-based training was performed within a safe heart rate range, with an average pulse of approximately 80 bpm across all training sessions.

Lastly, participants in the VR-related training were asked about the helpfulness of VR gaming in maintaining mental activity. A total of 84.3% of responders indicated a positive impact, with 21.1% selecting “definitively yes” and 63.2% “probably yes”. Meanwhile, 15.7% of participants answered, “hard to say”. Notably, no one selected “probably no” or “definitely no”.

## 4. Discussion

The aim of this study was to evaluate the role of VR-related physical activity using the commercial music game “*Beat Saber*” in improving eye–hand coordination and attention concentration in the elderly. Our results showed that a 4-week VR-based training effectively enhanced EHC and AC in seniors. Improvements were also observed in the control group, who participated in a standard training program at the Senior House. However, the analyzed parameters increased three times more in the VR group than in the control group. Virtual reality (VR) technology, as an integral part of current and future advancements, plays a crucial role in promoting healthy ageing and offering alternative or improved care and welfare solutions for the elderly. To date, several studies have analyzed the impact of VR-based interventions on the seniors’ physical and mental functionality, as well as their quality of life (QoL). Thus, a systematic review and meta-analysis by Peng et al. (2024) [23] concluded that exergames in VR positively influence physical function, cognition, and depression among residents of nursing homes, regardless of their functional status. VR-related exergaming has been shown to benefit both physically fit individuals and those with cognitive impairments [23]. Additionally, a randomized pilot study with an observational approach demonstrated that VR positively affects information processing speed, working memory, and mood in seniors [24]. Furthermore, studies suggest that VR gaming has a greater impact on balance maintenance compared to traditional exercises or non-interventional training [25,26]. Notably, some authors emphasize the effectiveness of games that incorporate motion capture feedback mechanisms [27]. Within this context, it is important to highlight the crucial role of EHC in balance control. Older individuals with weaker EHC often experience greater difficulties in maintaining balance and performing daily activities [28]. Therefore, our study aligns with these findings by examining the potential of VR-related training in improving EHC.

In our analysis, aside from the group-level results demonstrating significant improvement in EHC following the VR intervention, individual cases were also considered. Among participants in the VR group, 95% showed improvement in their EHC and AC efficiency. On the other hand, 21.1% of participants in the control group exhibited a deterioration in EHC and AC when compared to their pre-intervention test results. These findings suggest that VR-based interventions may be more effective than standard activities in enhancing EHC and AC. The results confirm the potential of VR training not only to improve coordination efficiency but also to help seniors maintain their coordination and perceptual skills at a higher level. While improving current efficiency is crucial, maintaining it at the existing level is also a significant achievement [12,15,16,17,18,19], especially given that EHC, AC, and goal-oriented movement, such as reaching, play a key role in daily life activities but tend to decline with age [29]. Nevertheless, a positive effect of the 4-week standard training was observed in approximately 78% of controls, which warrants further investigation. In such a way, it cannot be excluded that the improvement in EHC and AC in the control group may be due to a learning effect from repeated testing. Similar observations were reported by Rutkowski et al. (2024) in a study involving younger individuals (a student group) [30], where participants achieved better results in later test trials, indicating a learning effect. In this study, in both the control and VR groups, test performance improved in the second trial compared to the first, with further improvement observed in the third trial. However, a few individuals, especially in the VR group, showed lower scores in the third trial 3, possibly due to fatigue of seniors during the test. It is worth noting that participants underwent the EHC assessment immediately after completing a 20-min VR exergaming session. Consequently, reduced concentration and/or increased fatigue cannot be ruled out as factors affecting their performance. To mitigate this, future studies should consider implementing a short break between VR gaming and the EHC test.

The break, as well as potential fatigue during gaming, corresponded with the high levels of engagement observed among seniors during the game. This phenomenon was confirmed by the results measuring the game’s effectiveness. It was analyzed through the count of “good cuts” and an overall upward trend was observed throughout the sessions. A notable decline in performance occurred in session 4; however, scores still remained higher than in session 1. This temporary drop in results appears to be linked to a decline in well-being reported by a significant percentage of participants on that particular day. On this occasion, participants exhibited reduced engagement and concentration, which could be attributed to decreased alertness, an essential factor in maintaining performance. This observation was supported by both subjective reports from participants and objective performance measures [31]. Despite this minor disruption and two seniors reporting discomfort, the overall 4-week intervention in the senior group was successful. This outcome is particularly encouraging given the advanced age of the participants. Our study involved a group with a mean age of 87.2 years, an oldest-old population rarely examined in similar research. Importantly, the VR game proved to be safe, as no significant symptoms of cybersickness were reported, and the average heart rate during gaming remained at 80 bpm. This HR aligns with the American Heart Association’s recommended level for moderate-intensity exercise (range between 50 and 70% of an individual’s maximum heart rate, depending on age) [32]. Additionally, the positive results were achieved in a seated position, which is preferred for seniors as it provides greater safety compared to standing.

The safety of VR is particularly important in the context of its effectiveness. The intervention placed significant physical demands on participants, providing a multi-component training program. This aligns with the statement of Campo-Prieto et al. (2022), who emphasized that programs addressed to the seniors should integrate both physical and cognitive training to achieve comprehensive results [33]. Consistent with this approach, previous studies have shown that players of action games tend to have faster reaction time and better eye–hand coordination [34]. While it is already known that commercial games, including “*Beat Saber*”, positively impact physical and cognitive functions, including eye–hand coordination, these results have predominantly been observed in the younger age group [30,35,36]. In our study, “*Beat Saber*” training incorporated these key elements, offering improvements in EHC, AC, and, incidentally, also a reduction in reaction time. Notably, enhancing reaction time requires a combination of both physical and cognitive factors [37]. This is especially important for populations with declining functional abilities, such as seniors and individuals with conditions like Parkinson’s disease, as improved reaction time is associated with a reduced risk of falls in these groups [38]. It is important due to the fact that among the many challenges associated with an ageing society are those related to falls and their consequences [39]. Commercial VR games hold great potential in senior therapy, and the implementation of immersive, non-interactive VR interventions appears feasible for the elderly [33]. For example, use of VR therapy as a beneficial addition to the rehabilitation process of older people is observed following hip and knee arthroplasty [40]. However, VR-based training effectiveness is rather determined by an individual’s physical and cognitive condition [41]. Further efforts are needed to enhance VR accessibility for seniors, including additional research to confirm its effectiveness, optimization of game mechanics, and standardization of therapy programs. VR presents numerous possibilities and is a promising tool for rehabilitation. Our study provides hope that it can be successfully utilized across all age groups, including the oldest-old population.

Commercial VR games hold great potential in senior therapy, and the implementation of immersive, non-interactive VR interventions appears feasible for the elderly [42]. However, their effectiveness is rather determined by an individual’s physical and cognitive condition [41]. Further efforts are needed to enhance VR accessibility for seniors, including additional research to confirm its effectiveness, optimization of game mechanics, and standardization of therapy programs. VR presents numerous possibilities and is a promising tool for rehabilitation. Our study provides hope that it can be successfully utilized across all age groups, including the oldest-old population.

## 5. Limitations

Some limitations were identified in our study. The sample size was relatively small, consisting of only 38 participants, and despite randomization, the control and VR groups differed in their initial state. A notable strength of our study is the advanced mean age of the participants (87 years), which is rarely observed in similar research. On the other hand, the findings should be validated in an independent, larger cohort of seniors, ideally matched on key parameters before intervention. It should be noted that our study focused on a single commercial VR game, “*Beat Saber*”, and the intervention was relatively short (lasting 4 weeks and included 12 training sessions). While future studies might consider a longer duration, this could reduce adherence and the number of participants completing the entire program. Additionally, in future research, it should be considered to incorporate a broader range of VR games. These optimizations may provide more comprehensive and generalizable findings.

## 6. Conclusions

In conclusion, a 4-week intervention (12 sessions, 3 times a week) using an immersive, music-based VR game positively impacts cognitive functions such as eye-hand coordination, attention, concentration, and reaction time in the elderly. Moreover, it proves to be a safe form of physical activity.

## Figures and Tables

**Table 1:** Characteristics of the study groups.

Groups (N)	Age (Years)	Sex (Women/Men)	Weight (kg)	Height * (cm)	BMI (kg/m^2^)
All cases (38)	87.2 (±6.3)	29/9	68 (±14.5)	157.4 (±10.2)	27.7 (±6.4)
VR group (19)	88.0 (±6.4)	14/5	70.8 (±8.3)	161.4 (±8.3)	27.2 (±6.6)
Control group (19)	86.4 (±6.3)	15/4	65.1 (±12.2)	153.5 (±10.6)	28.2 (±6.4)

N, number of individuals; VR, virtual reality; BMI, body mass index; * significant differences in comparison between the VR group and control group (*p* < 0.05); all values are presented as means ± standard deviations (SD).

## Data Availability

The data presented in this study are available on request from the corresponding author. The data are not publicly available due to the protection of research participant privacy.
